# Synthesis and Antioxidant Activity of Alkyl Nitroderivatives of Hydroxytyrosol

**DOI:** 10.3390/molecules21050656

**Published:** 2016-05-18

**Authors:** Elena Gallardo, Rocío Palma-Valdés, Beatriz Sarriá, Irene Gallardo, José P. de la Cruz, Laura Bravo, Raquel Mateos, José L. Espartero

**Affiliations:** 1 Department of Organic and Pharmaceutical Chemistry, Faculty of Pharmacy, University of Seville, 41012 Seville, Spain; elegm@us.es (E.G.); xiopalma@yahoo.es (R.P.-V.); irenegalmar@hotmail.com (I.G.); 2 Department of Metabolism and Nutrition, Institute of Food Science, Technology and Nutrition (ICTAN), Spanish National Research Council (CSIC), 28040 Madrid, Spain; beasarria@ictan.csic.es (B.S.); lbravo@ictan.csic.es (L.B.); 3 Department of Pharmacology, Faculty of Medicine, University of Malaga, 29071 Malaga, Spain; jpcruz@uma.es

**Keywords:** nitrocatechol, alkyl nitrohydroxytyrosyl derivatives, hydroxytyrosol, antioxidant activity, Parkinson’s disease

## Abstract

A series of alkyl nitrohydroxytyrosyl ether derivatives has been synthesized from free hydroxytyrosol (HT), the natural olive oil phenol, in order to increase the assortment of compounds with potential neuroprotective activity in Parkinson’s disease. In this work, the antioxidant activity of these novel compounds has been evaluated using Ferric Reducing Antioxidant Power (FRAP), 2,2′-azinobis(3-ethylbenzothiazoline-6-sulfonic acid) diammonium salt (ABTS), and Oxygen Radical Scavenging Capacity (ORAC) assays compared to that of nitrohydroxytyrosol (NO_2_HT) and free HT. New compounds showed variable antioxidant activity depending on the alkyl side chain length; compounds with short chains (2–4 carbon atoms) maintained or even improved the antioxidant activity compared to NO_2_HT and/or HT, whereas those with longer side chains (6–8 carbon atoms) showed lower activity than NO_2_HT but higher than HT.

## 1. Introduction

**Figure 1:**
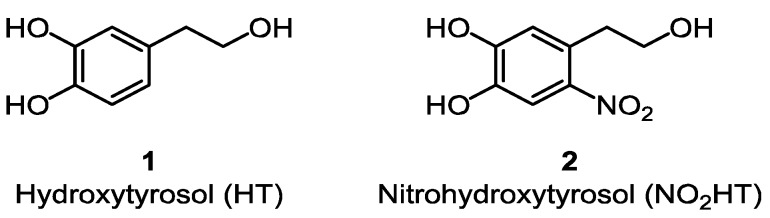
Chemical structures of parent compounds (**1**) and (**2**).

Adherence to the Mediterranean Diet has been associated with reduced risk of suffering from several pathological conditions including cardiovascular, cerebrovascular and neurodegenerative diseases [1]. Many studies conclude that polyphenols present in olive oil are responsible, among other compounds, for these effects due mainly to their potent antioxidant activities [2,3,4]. The main phenolic compounds in olive oil are free hydroxytyrosol (HT, **1**) (Figure 1) and its secoiridoid derivatives. In this sense, the European Food Safety Authority (EFSA) has recently released a health claim concerning phenolic compounds in olive oil and their ability to protect low-density lipoprotein from oxidative damage [5].

A wide range of associated biological activities have been described for HT, such as the capacity to scavenge free radical species [6] as well as to regulate antioxidant enzyme activity [7]. These properties have in common their role against oxidative stress, which underlies neurodegenerative diseases such as Parkinson’s disease (PD).

However, the highly polar nature of HT reduces its solubility in lipids, and, thus, efforts have focused on the syntheses of HT derivatives with an enhanced hydrophilic/lipophilic balance [8]. In fact, alkylation of HT with side chains of variable length has given rise to alkyl lipophilic HT ethers [9], which have shown enhanced antioxidant activity in comparison to their precursor, if the aliphatic chain length was up to 6–8 carbon atoms [9,10]. This result was in good accordance with the well-known cut-off effect, previously described in other lipophilic derivatives of polyphenols [11,12].

Most efforts in the treatment of PD have been directed at developing novel drugs with protective activity on the central nervous system. For years, in the clinical treatment of PD, nitrocatechols have been used in combination with levodopa [13,14], as the nitrocatechol ring seems to play an essential role in the effective catechol *orto*-methyl transferase (COMT) inhibition. Moreover, it is known that reactive oxygen species are closely related to neurodegeneration; therefore, if, in addition, the novel compounds present antioxidant activity, their therapeutic potential is larger [15].

In view of all the above, new lipophilic nitroderivative compounds derived from HT, acyl nitrohydroxytyrosyl derivatives, have been synthetized via nitrohydroxytyrosol (NO2HT, **2**), and their antioxidant potential has been tested [16]. Some compounds of this series have been studied, having shown a significant effect on the COMT activity in both striatal tissue [17] and extracellular dialysate levels in rats [18].

**Scheme 1:**
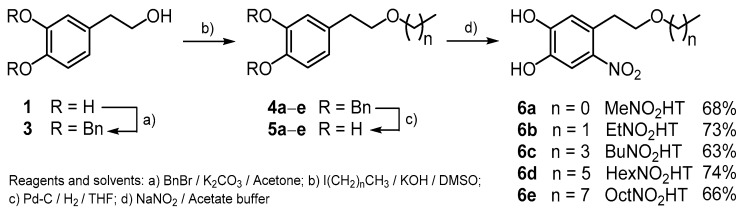
Synthetic route for the preparation of the hydroxytyrosol (HT) nitroderivatives (**6a**–**e**).

In the present work, the synthesis of alkyl ethers nitroderivatives of HT (**6a**–**e**), with side chain lengths from one to eight carbon atoms (Scheme 1) is presented, as a second family of lipophilic nitroderivatives of HT, and their antioxidant activity is evaluated using Reducing Antioxidant Power (FRAP), 2,2′-azinobis(3-ethylbenzothiazoline-6-sulfonic acid) diammonium salt (ABTS), and Oxygen Radical Scavenging Capacity (ORAC) assays.

## 2. Results

In the present work, the synthesis of certain alkyl NO_2_HT ethers with variable side chain lengths (**6b**–**e**) is presented together with the evaluation of their antioxidant activity, which has been measured using complementary methods (FRAP, ABTS, and ORAC) and comparatively studied against results previously published for NO2HT [16] and HT [19].

### 2.1. Preparation and Characterization of Alkyl Nitrohydroxytyrosyl Ethers (***6a***–***e***)

The new compounds were obtained starting from HT recovered from olive oil wastewater (OOWW) following a four-step process (Scheme 1). Pure HT (**1**) was transformed into its known dibenzyl derivative (**3**) [20] by reaction with benzyl bromide/potassium carbonate in acetone. Alkylation of the free alcoholic group with the corresponding alkyl iodides and further hydrogenolytic cleavage of the protecting Bn groups yielded the alkyl hydroxytyrosyl ethers (**5a**–**e**), as previously described [9]. The desired alkyl NO2HT ethers (**6a**–**e**) were finally obtained in good yields by nitration using sodium nitrite in acetate buffer (Scheme 1) and subsequent purification by column chromatography. It is noteworthy that initially in this purification step a great loss of product was observed, possibly due to its retention by the stationary phase used (silica gel), greatly reducing the chemical yield of the whole process. In order to overcome this point, other inert substances were tested as a column stationary phase (alumina, C_18_) without success. However, the addition of a small amount of formic acid (1%) to the eluent used in each case (different hexane/diethyl ether mixtures) partially solved this drawback and the yield was increased.

Synthetic NO_2_HT ethers (**6a**–**e**) were characterized by Nuclear Magnetic Resonance (NMR) and High-Resolution Mass Spectrometry (HR-MS). ^1^H- and ^13^C-NMR chemical shifts (Table 1 and Table 2, respectively) were unequivocally assigned for each derivative by 2D heteronuclear correlation experiments (Hetero Single Quanta Correlation -HSQC- and Hetero Multiple Bond Correlation -HMBC- spectra) and were in good agreement with the proposed structures. In the aromatic proton region of all the new synthesized compounds (**6a**–**e**) (Table 1), only two signals are observed and could be easily assigned to H_4_ and H_7_, by analogy with free NO_2_HT (**2**) [16]. These signals remained virtually constant in their chemical shift values, confirming that the nitro group has been well incorporated in all compounds at position 8. This pattern contrasts with that of HT (**1**), in which a third resonance is observed in the aromatic region, corresponding to H_8_.

These data are corroborated in Table 2, which shows a shift downfield (Δδ ≈ +9–10 ppm) in the C_1_ signal of compounds **6a**–**e** as compared with **2**, due to the presence of the alkyl chain in this position. In addition, a slight modification of C_2_ resonance, due to the β-shielding effect produced by the presence of such substituent in compounds **6a**–**e,** is also observed. Finally, it is noteworthy that the δ value for the signal assigned to C_8_, which carries the nitro group, is almost constant throughout the series of synthesized derivatives ethers, and well away (Δδ ≈ +20 ppm) from that of **1**, that does not present the NO_2_ group in its structure.

In addition, the study of the molecular ion for compounds **6a**–**e** by HRMS spectrometry allowed for confirmation of the calculated molecular masses and elemental compositions for each compound, with a mass deviation ranging between 0.8 and 2.5 ppm (see Materials and Methods).

Lipophilicity of alkyl NO2HT ethers **6a**–**e** was expressed by the theoretical (LogPtheor) partition coefficients and compared to those of their precursors (**1** and **2**) (Table 3). NO_2_HT (**2**) showed the highest polar nature and HT (**1**) was the second most hydrophilic compound amongst all the evaluated compounds. Amongst new synthesized compounds (**6a**–**e**), the lipophilicity was progressively increasing with the length of the alkylic chain in a linear way.

### 2.2. Antioxidant Activity Evaluation of Alkyl NO2HT Ethers (***6b***–***e***)

**Figure 2:**
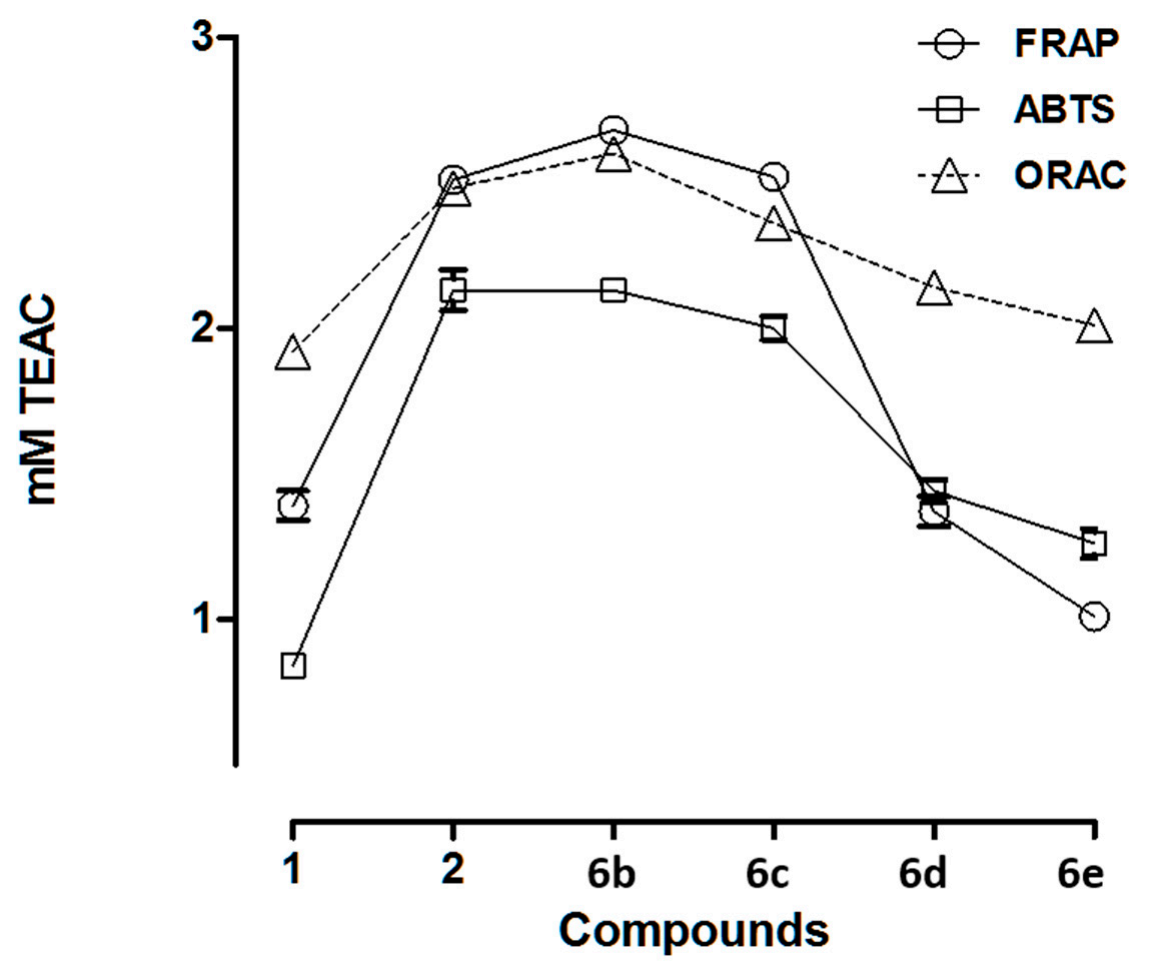
Antioxidant activity of from HT (**1**), NO2HT (**2**), and alkyl nitrohydroxytyrosyl ethers (**6b**−**e**) obtained using the FRAP, ABTS, and ORAC assays.

Results of the reducing power of new compounds (**6b**–**e**) obtained using the FRAP assay and the radical scavenging activity using ABTS and ORAC assays in comparison to free HT (**1**) and NO_2_HT (**2**) are summarized in Table 3 and Figure 2. Data are expressed as millimolar TEAC (Trolox equivalent).

#### 2.2.1. FRAP Assay

The antioxidant activity of the new alkyl nitroderivatives (**6b**–**e**) varied depending on the length of the alkyl side chain. In comparison to NO_2_HT (**2**), ethyl (**6b**) and butyl (**6c**) NO_2_HT ethers presented the same or higher antioxidant activity, whereas the more lipophilic hexyl (**6d**) and octyl (**6e**) derivatives showed lower activity, specifically that of **6e** corresponded to HT (**1**).

#### 2.2.2. ABTS Assay

The radical scavenging activities of the new compounds showed a similar trend to the reducing activity. In this sense, among the nitro derivatives, the compounds with shorter side chain, *i.e.*, ethyl (**6b**) and butyl (**6c**) NO_2_HT ethers, showed higher antioxidant activity. In contrast, the rest of the tested nitro compounds (**6d** and **6e**) presented lower antioxidant capacity than NO_2_HT (**2**) but higher than that of HT (**1**).

#### 2.2.3. ORAC Assay

The oxygen radical scavenging capacities of alkyl NO_2_HT ethers were in agreement with the results from ABTS analysis. The highest activity observed corresponded to ethyl NO_2_HT ether (**6b**) followed by the rest of the tested nitro derivatives, but always higher than HT (**1**).

## 3. Discussion

The results of a previous study on acyl NO_2_HT derivatives [16], prompted us to synthetize a new series, alkyl NO_2_HT derivatives, with small and medium side chain length (**6b**–**e**). According to Trujillo *et al.* [16], the length of the acyl side chain determined the antioxidant activity of the compounds, so that shorter chains maintained or even enhanced the antioxidant activity compared to NO_2_HT, but derivatives with longer side chain (>8 carbon atoms) showed a significantly lower antioxidant activity compared to NO_2_HT, even HT. Similarly, in the present study, the shorter alkyl side chains (from two to four carbon atoms) enhanced or maintained the antioxidant activity of NO_2_HT, whereas longer alkyl side chains (six and eight carbon atoms) showed lower antioxidant activity. All the synthetized compounds (**6b**–**e**) showed higher activity than HT, except for the reducing activity of **6e** determined by FRAP assay, which was slightly lower than HT. These results pointed out that the nitro group in the catecholic ring of HT positively affects the antioxidant activity of the compound, in accordance with the antioxidant activity of acyl NO_2_HT derivatives described by Trujillo *et al.* [16]. The higher antioxidant activity of NO_2_HTy compared to HT is also in agreement with the stabilization of the phenoxy radical with electro-donating substituents at positions *ortho* described by Pokorny [22] and Chimi [23]. Furthermore, the decrease in the antioxidant activity associated with the longer length of the acyl side chain could be due to steric hindrance. However, this disagrees with the polar paradox that states that polar antioxidants are more active in bulk lipids than their nonpolar counterparts, whereas nonpolar antioxidants are more effective in oil-in-water emulsions than their polar homologs [24]. The present results indicate that increased hydrophobicity does not always lead to increased antioxidant efficacy in non-fat environments, in accordance with previous results observed with alkyl hydroxytyrosyl ethers [9], and other lipophilic HT derivatives such as nitrohydroxytyrosyl esters [16], homovanillyl esters [21], as well as chlorogenate esters [25], and rosmarinate esters [26], among others.

Moreover, a nonlinear association between biological activity and the lipophilic nature of homologous series of molecules had already been described in different cell lines; ester derivatives of gallic acid were cytotoxic in L1210 leukemia cells [27], hydroxytyrosyl ethers showed antiplatelet effects in blood cells from humans [28] and rats [29]. Additionally, cytotoxic activity of hydroxytyrosyl alkyl ether derivatives against A549 lung cancer cells and MRC5 nonmalignant lung fibroblasts has been recently described [30]. In all of the aforementioned studies, biological activity increased up to medium length of the acyl or alkyl side chain, whereas the most lipophilic compounds showed lower biological activities. This nonlinear phenomenon was coined by Laguerre *et al.* [25] as the cut-off effect, and it relates the lower activity of the lipophilic compounds, and therefore higher molecular weight, than hydrophilic compounds due to their reduced mobility and self-aggregation phenomena or internalization in the organic phase, having been observed in both biological and physicochemical systems [31].

When the antioxidant activity of alkyl NO_2_HT compounds were compared to that of acyl NO_2_HT derivatives [16], between compounds with the same side chain length, the alkyl series were slightly less active than the acyl. However, the influence of the chemical bond nature (acyl *vs.* alkyl) on the antioxidant activity was lower than that of the side chain length and, therefore, the lipophilic nature of the compound. The chemical nature of the side chain substituent is emerging as an important factor in the biological activity of these kinds of compounds. Accordingly, structure-activity relationship studies (SAR) have demonstrated their influence on COMT activity pointing out that, although the nitrocatechol structure was mainly responsible for the “anchoring” of the inhibitor to the enzyme active site, variations in the side chain substituent exert a profound influence on both the peripheral selectivity and duration of COMT activity [14]. Indeed, the alkyl nitroderivatives NO_2_HT ether as well as its analogous NO_2_HT acetate have shown the capacity to inhibit brain COMT activity [17,18]. Additionally, all efforts in the treatment of Parkinson's disease are directed towards the development of novel drugs that offer neuroprotection by having various central nervous system targets [32].

Considering all the above mentioned, it may be gathered that alkyl lipophilic nitroderivatives with shorter chains present some interesting features as free radical scavenging and reducing activity to become in (promising) biological compounds with a broad pharmacological potential.

## 4. Materials and Methods

### 4.1. Materials

All solvents and reagents were of analytical grade unless stated otherwise. 6-Hydroxy-2,5,7,8tetramethylchroman-2-carboxylic acid (Trolox), anhydrous sodium sulfate, hexane, tetrahydrofuran (THF), diethyl ether, potassium persulfate, methanol, acetone, sodium nitrite, hydrogen chloride, iron (III) chloride hexahydrate, sodium hydrogen phosphate, potassium dihydrogen phosphate, acetic acid, sodium acetate trihydrate, hexadeuterated dimethyl sulfoxide (DMSO-*d*_6_) were from Aldrich (Madrid, Spain). Fluorescein, methylated β-cyclodextrin (RMCD), 2,2′-azinobis(3ethylbenzothiazoline-6-sulfonic acid) diammonium salt (98%) (ABTS), 2,2′-azobis(2amidinopropane) dihydrochloride (AAPH), and 2,4,6-tri-(2-pyridyl)-1,3,5-triazine (TPTZ) were from Sigma (Madrid, Spain). HT was recovered with 95% purity from OOWW [33] and further purified by column chromatography.

NMR spectra were recorded on a Bruker AVANCE 500 spectrophotometer (Bruker, Madrid, Spain) operating at 500.13 MHz (^1^H) and 125.75 MHz (^13^C). Chemical shifts are given in parts per million, with the residual solvent signals (2.49 ppm for ^1^H and 39.5 ppm for ^13^C) as references. Samples were dissolved (10−20 mg/mL) in DMSO-*d*_6_, and spectra were recorded at 303 K. High-resolution CI mass spectra (HRMS) were obtained on a Micromass AUTOSPECQ spectrometer (Micromass, Madrid, Spain). Theoretical values of partition coefficient (Log P_theor_) of the new synthesized compounds were determined using the ChemBioDraw Ultra software (version14.0) (CambridgeSoft).

### 4.2. Synthetic Procedures

#### 4.2.1. Synthesis of Hydroxytyrosyl Ethers (**5a**–**e**)

Compounds **5a**−**e** were obtained as previously described in Madrona *et al.* [9] Briefly, benzyl bromide (1.4 mL, 11.8 mmol) and potassium carbonate (2.9 g, 20.8 mmol) were added to a solution of pure HT (**1**, 0.8 g, 5.2 mmol) in dry acetone (25 mL), and the resulting mixture was heated to reflux for 24 h. The obtained suspension was filtered and concentrated to yield a crude residue, which was further purified by column chromatography, using a mixture (1:2) of diethyl ether/hexane as eluent, to yield **3**. A mixture of **3** (334 mg, 1 mmol), KOH (335 mg) and the corresponding alkyl iodide (3 mmol) in methyl sulfoxide (12 mL) was stirred at room temperature until completion of reaction (TLC). 25 mL of 3 M HCl was then added and the mixture extracted with CHCl_3_ (3 × 25 mL). The organic phase was washed with 2% NaHSO_3_ (25 mL) and water (25 mL), dried over Na_2_SO_4_, filtered and evaporated. Compounds **4a**–**e** were purified by flash column chromatography over silica gel. Finally, palladium over charcoal (Pd-C) was added to a solution of the corresponding ether (**4a**–**e**, 1 mmol) in THF (20 mL) and the mixture was hydrogenated at 4 bar with magnetic stirring. After 24 h at room temperature, the catalyst was filtered off and solvent was evaporated in vacuum, yielding the desired compound in each case (**5a**–**e**) that was purified by column chromatography.

#### 4.2.2. General Method of Nitration

The corresponding ether (**5a**–**e**, 1 mmol) was added to 0.1 M acetate buffer (pH 3.8) (200 mL) followed by sodium nitrite (138 mg, 2 mmol). After 30 min at room temperature, the mixture was extracted with ethyl acetate (6 × 50 mL) and the combined organic layers were dried over anhydrous sodium sulfate, filtered and evaporated to give a residue that was further purified by column chromatography (different hexane/diethyl ether mixtures as eluents) to obtain the corresponding pure alkyl nitrohydroxytyrosyl ether (**6a**–**e**).

*Data for methyl nitrohydroxytyrosyl ether* (**6a**): 68% yield. Obtained as a white solid: mp 152–154 °C. NMR (500 MHz, DMSO-*d*_6_ δ ppm 10.06 (bs, 2H, 2 phenolic OH’s), 7.45 (s, 1H, H7), 6.75 (s, 1H, H4), 3.48 (t, *J* = 6.6 Hz, 2H, H1), 3.21 (s, 3H, H1′), 2.99 (t, 2H, H2); ^13^C-NMR (125 MHz, DMSO-*d*_6_ δ ppm 151.0 (C5), 143.9 (C6), 139.6 (C8), 127.3 (C3), 118.3 (C4), 112.0 (C7), 71.7 (C1), 57.8 (C1´), 32.5 (C2); HRMS (CI) *m*/*z* calcd for C_9_H_11_NO_5_ [M]^+^ 213.0637, found 213.0632 (2.5 ppm). Log P_theor_ 1.47.

*Data for ethyl nitrohydroxytyrosyl ether* (**6b**): 73% yield. Obtained as a white solid: mp 94–96 °C ^1^H-NMR (500 MHz, DMSO-*d*_6_ δ ppm 10.11 (bs, 2H, 2 phenolic OH’s), 7.44 (s, 1H, H7), 6.76 (s, 1H, H4), 3.51 (t, *J* = 6.7 Hz, 2H, H1), 3.39 (c, *J* = 7.0 Hz, 2H, H1′), 2.99 (t, *J* = 6.7 Hz, 2H, H2), 1.06 (t, *J* = 7.0 Hz, 3H, H2′); ^13^C-NMR (125 MHz, DMSO-*d*_6_ δ ppm 151.0 (C5), 143.9 (C6), 139.8 (C8), 127.3 (C3), 118.3 (C4), 112.0 (C7), 69.6 (C1), 65.2 (C1′), 32.8 (C2), 15.0 (C2′); HRMS (CI) *m*/*z* calcd for C_10_H_14_NO_5_ [M + H]^+^ 228.0872, found 228.0867 (2.2 ppm). Log P_theor_ 1.84.

*Data for n-butyl nitrohydroxytyrosyl ether* (**6c**): 63% yield. Obtained as a syrup: ^1^H-NMR (500 MHz, DMSO-*d*_6_ δ ppm 10.2 (bs, 2H, 2 phenolic OH’s), 7.44 (s, 1H, H7), 6.75 (s, 1H, H4), 3.51 (t, *J* = 6.8 Hz, 2H, H1), 3.33 (t, *J* = 6.5 Hz, 2H, H1´), 2.99 (t, *J* = 6.8 Hz, 2H, H2), 1.42 (q, 2H, H2′), 1.26 (m, 2H, H3′), 0.84 (t, *J* = 7.0 Hz, 3H, H4′); ^13^C-NMR (125 MHz, DMSO-*d*_6_ δ ppm 151.0 (C5), 143.8 (C6), 139.8 (C8), 127.4 (C3), 118.4 (C4), 112.0 (C7), 69.8 (C1), 69.6 (C1′), 32.8 (C2), 31.2 (C2′), 18.8 (C3´), 13.7 (C4′); HRMS (CI) *m/z* calcd for C_12_H_18_NO_5_ [M + H]^+^ 256.1185, found 256.1183 (0,8 ppm). Log P_theor_ 2.75.

*Data for n-hexyl nitrohydroxytyrosyl ether* (**6d**): 74% yield. Obtained as a syrup: ^1^H-NMR (500 MHz, DMSO-*d*_6_) δ ppm 10.02 (bs, 2H, 2 phenolic OH’s), 7.44 (s, 1H, H7), 6.75 (s, 1H, H4), 3.50 (t, *J* = 6.7 Hz, 2H, H1), 3.32 (t, *J* = 6.6 Hz, 2H, H1′), 2.99 (t, 2H, H2); 1.43 (q, 2H, H2′), 1.26–1.20 (m, 6H, H3′-H5′), 0.83 (t, *J* = 7,0 Hz, 3H, H6′); ^13^C-RMN (125 MHz, DMSO-*d*_6_) δ ppm 151.0 (C5), 143.8 (C6), 139.8 (C8), 127.4 (C3), 118.4 (C4), 112.0 (C7), 69.9 (C1), 69.8 (C1′), 32.7 (C2), 31.0 (C4′), 29.1 (C2′), 25.2 (C3′), 22.0 (C5′), 13.8 (C6′); HRMS (CI) *m*/*z* calcd for C_14_H_22_NO_5_ [M + H]^+^ 284.1498, found 284.1494 (1,4 ppm). Log P_theor_ 3.66.

*Data for n-octyl nitrohydroxytyrosyl ether* (**6e**): 66% yield. Obtained as a white solid: mp 84–86 °C; ^1^H-NMR (500 MHz, DMSO-*d*_6_) δ ppm 10.02 (bs, 2H, 2 phenolic OH’s ), 7.44 (s, 1H, H7), 6.75 (s, 1H, H4), 3.50 (t, *J* = 6.7 Hz, 2H, H1), 3.32 (t, *J* = 6.6 Hz, 2H, H1′), 2.99 (t, 2H, H2); 1.43 (q, 2H, H2′), 1.26–1.20 (m, 10 H, H3′-H7′), 0.84 (t, *J* = 7.0 Hz, 3H, H8′); ^13^C-RMN (125 MHz, DMSO-*d*_6_) δ ppm 151.0 (C5), 143.8 (C6), 139.7 (C8), 127.4 (C3), 118.3 (C4), 112.0 (C7), 69.9 (C1), 69.8 (C1′), 32.7 (C2), 31.2 (C6′), 29.1 (C2′), 28.7 (C4′), 28.6 (C5′), 25.6 (C3′), 22.0 (C7′), 13.9 (C8′); HRMS (CI) *m*/*z* calcd for C_16_H_26_NO_5_ [M + H]^+^ 312.1811, found 312.1808 (1.0 ppm). Log P_theor_ 4.57.

### 4.3. Antioxidant Activity Determinations

#### 4.3.1. Ferric Reducing Antioxidant Power (FRAP) Assay

The FRAP assay was carried out according to the procedure described by Pulido *et al.* [34]. The antioxidant potential of the synthesized compounds was estimated from their ability to reduce the ferric tripyridyltriazine (TPTZ-Fe(III)) complex to its stable ferrous form (TPTZ-Fe(II)). Briefly, the FRAP reagent contained 2.5 mL of a 10 mM TPTZ solution in 40 mM HCl, plus 2.5 mL of 20 mM FeCl_3_·6H_2_O and 25 mL of 0.3 M acetate buffer to a final pH of 3.6. This reagent was freshly prepared and warmed to 37 °C prior to use. Nine hundred microliters of FRAP reagent was mixed with 90 μL of distilled water and 30 μL of either a standard, methanol (as appropriate reagent blank), or a test sample (ranging from 50 to 400 μM for ethers with short (<6) alkyl chain and from 100 to 1000 μM for ethers with medium (≥6) alkyl chain). All compounds were dissolved in methanol. Once the mixture was shaken, readings at the absorption maximum at 595 nm were taken every 20 s, and the reaction was monitored up to 30 min at 37 °C, using a UV−visible Varian (Cary 50 BIO, Varian, Madrid, Spain) spectrophotometer equipped with a thermostatic autocell-holder. The reading at 30 min was selected in each case for the calculation of FRAP values. Methanol solutions of Trolox were used for calibration. The FRAP values are expressed as millimolar TEAC (Trolox equivalent,). All analyses were run in triplicate.

#### 4.3.2. ABTS Assay

The free radical scavenging capacity was measured using the ABTS discoloration method [35] with some modifications. The method is based on the capacity of different components to scavenge the ABTS radical cation (ABTS^•+^) compared to a standard antioxidant (Trolox). Briefly, ABTS was dissolved in a 2.45 mM potassium persulfate solution and stored in the dark at room temperature for 12–16 h, to set a 7 mM concentration of ABTS radical cation (ABTS^•+^) solution. The ABTS^•+^ stock solution was diluted with methanol to get an absorbance of 0.70 ± 0.02 at 730 nm. After the addition of 0.1 mL of sample dissolved in methanol (ranging from 50 to 400 μM for ethers up to butyl and from 100 to 500 μM for octyl), methanol as a blank, or Trolox standard to 3.9 mL of diluted ABTS^•+^ solution, absorbance readings were taken every 20 s at 30 °C over 6 min, using a UV−visible spectrophotometer. The percentage inhibition of absorbance was plotted against time, and the area under the curve (0–360 s) was calculated. Methanol solutions of known concentrations of Trolox were used for calibration. Results are expressed in millimolar TEAC (Trolox equivalent). Each value is the average of three determinations.

#### 4.3.3. Oxygen Radical Scavenging Capacity (ORAC) Assay

The oxygen radical scavenging capacity was measured by the lipophilic ORAC assay according to the method developed by Huang *et al.* [36] with some modifications. The assay is based on the fluorescence decay of a reference substance (fluorescein) after the addition of a peroxyl radical (AAPH), which acts as an initiator of the oxidative reaction. Nitroderivatives (**6b**−**e**) from 5 to 25 μM and Trolox standard (6.25, 12.5, 25, 50, 75 and 100 μM) were dissolved in 7% methylated βcyclodextrin (RMCD) in acetone/water (1:1, *v*/*v*) solution. Then, 25 μL of either Trolox, test sample, or solvent as blank was added to a 96-well microplate followed by the addition of 150 μL of fluorescein work solution (8.5 × 10^−5^ mM) prepared in 75 mM phosphate buffer (pH 7.4). The microplate reader (Bio-Tek, Winooski, VT, USA) was programmed to record every 2 min for 120 min at 485 and 528 nm excitation and emission wavelengths, respectively, the fluorescence after the addition of 30 μL of AAPH (153 mM) as peroxyl radical generator, which was also prepared in 75 mM phosphate buffer (pH 7.4). Trolox was used for calibration, and values are expressed as millimolar TEAC (Trolox equivalent). All analyses were run in quadruplicate.

### 4.4. Statistical Analysis

Results are expressed as means ± standard deviation of three measurements for the ABTS and FRAP assays and four determinations for the ORAC assay. Results were statistically studied by one-way analysis of variance (ANOVA) using the SPSS statistical package (version 20.0; SPSS, Inc., IBM Madrid, Spain). The level of significance was set at *p* < 0.05.

## 5. Conclusions

In conclusion, among the series of alkyl nitrohydroxytyrosyl ether derivatives that have been synthesized from natural olive oil phenol HT, compounds with short alkyl side chain lengths showed higher antioxidant activity, determined by FRAP, ABTS and ORAC assays, compared to HT, so that the longer the length of chain, the lower the antioxidant activity, in accordance with the cut-off effect.

## Figures and Tables

**Table 1:** ^1^H-NMR Data (500.13 MHz, hexadeuterated dimethyl sulfoxide DMSO-*d*_6_, 303 K) for compounds **1**, **2** and **6a**–**e** ^a^.

Position	1	2	6a	6b	6c	6d	6e
**Phenethyl Unit**							
1	3.49 (t) (*J*_1,2_ = 7.2)	3.56 (t) (*J*_1,2_ = 6.8)	3.48 (t) (*J*_1,2_ = 6.6)	3.51 (t) (*J*_1,2_ = 6.7)	3.51 (t) (*J*_1,2_ = 6.7)	3.50 (t) (*J*_1,2_ = 6.5)	3.50 (t) (*J*_1,2_ = 6.7)
2	2.52 (t)	2.90 (t)	2.99 (t)	2.99 (t)	2.99 (t)	2.99 (t)	2.99 (t)
4	6.57 (d) (*J*_4,8_ = 2.0)	6.75 (s)	6.75 (s)	6.76 (s)	6.75 (s)	6.75 (s)	6.75 (s)
7	6.60 (d) (*J*_7,8_ = 8.0)	7.43 (s)	7.45 (s)	7.44 (s)	7.44 (s)	7.44 (s)	7.44 (s)
8	6.42 (dd)						
**Alkyl Chain**							
1′			3.21 (s)	3.39 (q) (^3^*J* = 7.0)	3.33 (t) (^3^*J* = 6.5)	3.32 (t) (^3^*J* = 6.6)	3.22 (t) (^3^*J* = 6.6)
2′				1.06 (t)	1.42 (m)	1.43 (m)	1.43 (m)
3′					1.26 (m)	1.22 (m)	1.22 (m)
4′					0.84 (t) (^3^*J* = 7.0)		
5′							
6′						0.83 (t) (^3^*J* = 7.0)	
7′							
8′							0.84 (t) (^3^*J* = 7.0)

**^a^** Chemical shifts (δ, ppm) and coupling constants (*J*, Hz). Symbols: s, singlet; d, doublet; t, triplet; q, quartet; dd, double doublet; m, multiplet.

**Table 2:** ^13^C-NMR Chemical Shifts (ppm) (125.76 MHz, DMSO-*d*_6_, 303 K) for compounds **1**, **2** and **6a**–**e**.

Position	1	2	6a	6b	6c	6d	6e
**Phenethyl Unit**							
1	62.5	61.0	71.7	69.6	69.8	69.9	69.9
2	38.4	36.0	32.5	32.8	32.8	32.7	32.7
3	130.1	127.8	127.3	127.3	127.4	127.4	127.4
4	116.2	118.5	118.4	118.3	118.4	118.4	118.3
5	144.8	150.9	151.0	151.0	151.0	151.0	151.0
6	143.2	143.7	143.9	143.9	143.8	143.8	143.8
7	115.3	112.0	112.0	112.0	112.0	112.0	112.0
8	119.3	139.7	139.6	139.8	139.8	139.8	139.7
**Alkyl Chain**							
1′			57.8	65.2	69.6	69.8	69.8
2′				15.0	31.2	29.1	29.1
3′					18.8	25.3	25.6
4′					13.7	31.0	28.7
5′						22.0	28.6
6′						13.8	31.2
7′							22.0
8′							13.9

**Table 3:** Log P_theor_, reducing power, evaluated using the FRAP assay, and radical scavenging activity, determined using ABTS and ORAC assays, of hydroxytyrosol (**1**), nitrohydroxytyrosol (**2**), and alkyl nitrohydroxytyrosyl ethers (**6b**−**e**).

Compound	Log P_theor_	FRAP Assay (mM)	ABTS Assay (mM)	ORAC Assay (mM)
**1** *	0.96	1.39 ± 0.05 ^c^	0.84 ± 0.02 ^e^	1.92 ± 0.04 ^f^
**2** *	0.75	2.51 ± 0.04 ^b^	2.13 ± 0.07 ^a^	2.48 ± 0.04 ^b^
**6b**	1.84	2.68 ± 0.03 ^a^	2.13 ± 0.03 ^a^	2.60 ± 0.05 ^a^
**6c**	2.75	2.52 ± 0.04 ^b^	2.00 ± 0.04 ^b^	2.36 ± 0.04 ^c^
**6d**	3.66	1.37 ± 0.05 ^c^	1.44 ± 0.04 ^c^	2.14 ± 0.03 ^d^
**6e**	4.57	1.01 ± 0.03 ^d^	1.26 ± 0.05 ^d^	2.01 ± 0.03 ^e^

The antioxidant data represent the mean ± standard deviation for three determinations for FRAP and ABTS assays and four determinations for ORAC assay. Results are expressed as mM Trolox equivalent (TEAC, mM). All values within a column with different letters are significantly different, *p* < 0.05. * FRAP and ABTS values of HT (**1**) [21] and NO_2_HT (**2**) [16] have been previously published and are included for comparative purposes.

## Abbreviations

The following abbreviations are used in this manuscript:

AAPH: 2,2′-azobis(2-amidinopropane) dihydrochloride
ABTS: 2,2′-azinobis(3-ethylbenzothiazoline-6-sulfonic acid) diammonium salt
COMT: Catechol *orto*-Methyl Transferase
DMSO-*d*_6_: hexadeuterated dimethyl sulfoxide
EFSA: European Food Safety Authority
FRAP: Ferric Reducing Antioxidant Power
HMBC: Hetero Multiple Bond Correlation
HRMS: High-Resolution Mass Spectrometry
HSQC: Hetero Single Quanta Correlation
HT: Hydroxytyrosol
NMR: Nuclear Magnetic Resonance
NO2HT: Nitrohydroxytyrosol
OOWW: Olive Oil Wastewaters
ORAC: Oxygen Radical Scavenging Capacity
PD: Parkinson’s Disease
TEAC: Trolox Equivalent Antioxidant Capacity
TPTZ: 2,4,6-tri-(2-pyridyl)-1,3,5-triazine
